# Determinants of Emergency Department Length of Stay and the Mediation Effect of Disposition Among Injury Patients in South Korea: A Nationwide Retrospective Study

**DOI:** 10.3390/healthcare14040469

**Published:** 2026-02-12

**Authors:** Min-Seok Choi, Su-il Kim, Yun-Deok Jang

**Affiliations:** 1 Department of Health Administration, Yeungnam University College, Daegu 42415, Republic of Korea; dreebok@naver.com; 2 Department of Paramedicine, Yeungjin University, Daegu 41527, Republic of Korea; avantetop@hanmail.net; 3 Department of Paramedicine, Tongmyong University, Busan 48520, Republic of Korea

**Keywords:** emergency service, admission, transfer, severity of illness index

## Abstract

**Background/Objectives**: Emergency department length of stay (ED LOS) is a key indicator reflecting emergency department crowding, patient safety, and healthcare resource efficiency. Among injured patients, ED LOS may be prolonged depending on injury severity and disposition pathways (admission and inter-hospital transfer). This nationwide study using the Korean National Emergency Department Information System (NEDIS) aimed to (1) describe the distribution and determinants of ED LOS among injured patients and (2) quantify the mediating effects of disposition (admission and transfer) on the association between injury severity measured by the International Classification of Diseases-based Injury Severity Score (ICISS) and ED LOS. **Methods**: We analyzed NEDIS injury-related ED visit records collected from the date of IRB approval through 12 January 2026. We conducted a retrospective observational study using NEDIS data. Of 1,048,575 injury-related ED visits, 1,035,484 visits with valid ED LOS and eligible records were included after excluding missing key variables and implausible time values. ED LOS was calculated in minutes using arrival and departure timestamps. Injury severity was assessed using ICISS (primary: based on 15 diagnoses; sensitivity: based on 20 diagnoses). Determinants of ED LOS were evaluated using gamma regression with a log link. Disposition was categorized as discharge, admission, and inter-hospital transfer; admission and transfer were modeled as binary mediators. Causal mediation analyses estimated the average causal mediation effect (ACME), average direct effect (ADE), total effect, and proportion mediated. Multiple sensitivity analyses (outlier handling, missing-data approaches, alternative log-linear modeling, and EMS arrival subgroup analyses) assessed robustness. **Results**: The median ED LOS was 150 min (IQR 90–260). ED LOS differed substantially by disposition: 120 min for discharged patients, 420 min for admitted patients, and 360 min for transferred patients. Overall, 17.9% of visits had an ED LOS ≥ 6 h, and prolonged stays were concentrated among admitted (≥6 h: 55.0%) and transferred (≥6 h: 45.0%) patients. In gamma regression, a 0.05 decrease in ICISS (greater severity) was associated with longer ED LOSs in the unadjusted model (Ratio 1.34) and remained significant in the fully adjusted model (Ratio 1.12, 95% CI 1.11–1.13). Admission and transfer were strong determinants of ED LOS in the final model (ratios of 2.35 and 2.05, respectively). In mediation analyses, admission mediated 36.8% of the severity–ED LOS association (ACME 0.085; ADE 0.146), and transfer mediated 14.3% (ACME 0.033; ADE 0.198). Findings were consistent across sensitivity analyses. **Conclusions**: In this nationwide cohort of injured patients, ED LOS showed a right-skewed distribution, with prolonged stays concentrated in admission and transfer pathways. Injury severity (ICISS) was independently associated with longer ED LOS, and a substantial proportion of this association was mediated through admission and transfer. Reducing ED LOS among severely injured patients likely requires not only streamlining diagnostic and treatment processes but also system-level interventions targeting output-stage bottlenecks, including inpatient bed operations/boarding management and transfer coordination.

## 1. Introduction

Monitoring emergency department length of stay (ED LOS) is a key managerial indicator that reflects whether an emergency department can efficiently accommodate and process emergency patients, and within the conceptual framework of emergency department crowding that divides patient flow into input, throughput, output, ED LOS is regarded as a representative throughput metric [1]. As ED LOS increases, the space and staffing capacity needed to accept new emergency patients are consumed, resulting in delays in care and reduced timeliness of treatment; accumulating evidence has repeatedly shown that emergency department crowding is associated with poorer quality of care and adverse patient outcomes [2].

In the Republic of Korea, nationwide emergency department visit data are continuously accumulated through the National Emergency Department Information System (NEDIS), providing a foundation for comparing and evaluating ED LOS at the national level and for using these data as evidence to support policy interventions [3,4]. In an analysis of 25,578,263 NEDIS visits from 2018 to 2022, the median ED LOS was 2.1 h, and the proportion of prolonged ED LOS (≥6 h) was 12.6% [3]. A national trend report covering the same period also indicated that 90.3% of visits had an ED LOS <6 h, suggesting that although most patients leave the emergency department relatively quickly, a nontrivial proportion of prolonged-stay patients may substantially contribute to emergency department crowding and resource bottlenecks [4].

South Korean evidence further suggests that ED LOS may be linked to patient safety beyond merely representing “time spent” in the emergency department [5,6]. In a nationwide study of adult emergency department visits in Korea (2016–2017), the median ED LOS was 2.5 h; ED LOS was longer among patients who experienced in-hospital cardiac arrest, and prolonged ED LOS was reported as an independent risk factor for in-hospital cardiac arrest [5]. In addition, a nationwide NEDIS-based study of critically ill adult patients directly admitted to an intensive care unit reported a median ED LOS of 3.3 h, with 25.3% experiencing prolonged ED LOS (≥6 h); prolonged ED LOS remained significantly associated with increased in-hospital mortality after adjustment [6].

Prolonged ED LOS reflects not only clinical factors such as patient acuity but also system-level factors, including hospital bed availability, output-stage delays such as boarding, and processes related to inter-hospital transfer [1,2]. A nationwide cross-sectional study in Korea found that ED LOS increased substantially as the occupancy rate rose, and the increase was greatest among admitted patients, supporting the notion that output bottlenecks can structurally prolong ED LOS [7]. Moreover, evidence has suggested that emergency department overcrowding may also be associated with prehospital ambulance system performance, implying that the impact of ED LOS and crowding management can extend to the broader emergency medical system [8].

Inter-hospital transfer is a major pathway through which ED LOS can be prolonged, as it involves multistep processes such as identifying a receiving hospital, confirming resource availability, sharing clinical information, and preparing transport [2,9]. In a nationwide NEDIS-based study, patients undergoing double transfer had longer ED LOS than those with a single transfer (adult median, 189.0 min vs. 308.0 min), and a higher proportion of stays exceeding 12 h; lack of medical resources accounted for a substantial share of the reasons for double transfer [10]. Additionally, a single-center study implementing a boarding restriction protocol—designed to encourage bed assignment and transfer coordination within 24 h to manage boarding—reported reductions in emergency department occupancy and the proportion of stays exceeding 24 h, along with significant decreases in ED LOS and boarding time among admitted patients, supporting the potential effectiveness of output-stage interventions [9].

These domestic realities are also emphasized at the policy level [11,12,13]. The Emergency Medical Service Act stipulates that emergency medical institutions should take measures to minimize ED LOS and ensure timely admission for emergency patients requiring hospitalization, and it specifies that regional and local emergency medical centers must maintain the proportion of patients staying in the emergency department for more than 24 h below the standard set by the Ministry of Health and Welfare [11]. The Enforcement Rule of the Act further defines this standard as less than 5% per year, thereby formally establishing a national criterion for the annual proportion of ED stays exceeding 24 h [12]. The Ministry of Health and Welfare has also stated that periodic monitoring and feedback of the proportion of ED stays exceeding 24 h using NEDIS are intended to promote hospital-level quality improvement [13].

Notably, patients with injury (trauma) are at particularly high risk of prolonged ED LOS because their care pathways are complex, depending on the need for imaging, procedures, surgery, and linkage to intensive care; decisions regarding admission or transfer are frequent in this population [2,7,10]. In this context, injury severity is a key determinant of the intensity and breadth of diagnostic and therapeutic interventions, and the International Classification of Diseases-based Injury Severity Score (ICISS) has been widely used to estimate injury severity from diagnosis codes in administrative data settings [14].

Nevertheless, prior studies have largely focused on describing ED LOS levels and predictors or on evaluating associations between ED LOS and adverse outcomes, and few have quantified the effect of injury severity on ED LOS by decomposing it into a direct effect (greater testing and treatment driven by severity itself) and an indirect effect mediated through disposition pathways (admission and transfer) that accumulate output-stage delays [3,6,10]. Without separating and quantifying these pathways—whereby greater severity increases the likelihood of admission or transfer and delays related to bed assignment or securing a receiving hospital accumulate—it is difficult to distinguish the proportion of prolonged ED LOS that is potentially reducible through operational improvements from the portion that may be unavoidable [1,2].

Therefore, using raw NEDIS data, this study aims to characterize the distribution and determinants of ED LOS among injured patients and to quantify, within a causal mediation analysis framework, the mediation effect of disposition (admission and transfer) on the relationship between injury severity measured by ICISS and ED LOS [14,15].

By distinguishing the extent to which prolonged ED LOS is attributable to unavoidable severity-related care needs versus modifiable delays amenable to improvements in bed management and transfer systems, this study seeks to provide actionable evidence to reduce emergency department crowding and to enhance patient safety and the quality of emergency care.

## 2. Materials and Methods

### 2.1. Study Design

The study population comprised 1,048,575 injury-related ED visits recorded in NEDIS during the study period. The study period was defined as from the date of IRB approval to 12 January 2026. The primary objective was to characterize the distribution and determinants of emergency department length of stay (ED LOS) among injured patients presenting to the emergency department and to examine the mediation effect of disposition (admission and transfer) on the relationship between injury severity, measured by the International Classification of Diseases-based Injury Severity Score (ICISS), and ED LOS. Because this study used de-identified NEDIS data and involved no direct contact with participants, it was conducted after obtaining an exemption from review by the Institutional Review Board (IRB) (IRB No. 2026-01-021).

### 2.2. Study Population

**Figure 1:**
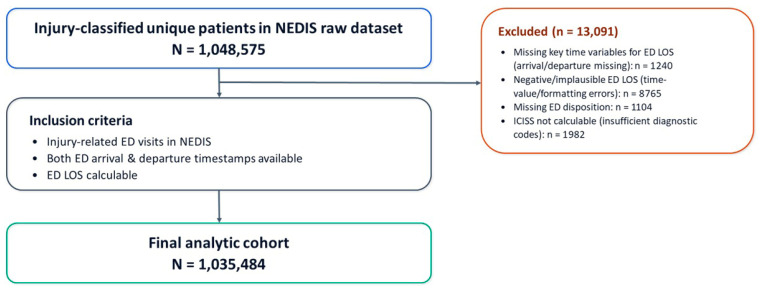
Study flow chart.

The study population comprised 1,048,575 patients whose emergency department (ED) visits were classified as injury-related in NEDIS. The study period extended from the date of Institutional Review Board (IRB) approval through 12 January 2026. Injury-related ED visit records were eligible for inclusion if they contained both ED arrival and departure timestamps, enabling calculation of ED length of stay (ED LOS). Records were excluded according to predefined data-cleaning rules when key time variables required to compute ED LOS (e.g., arrival or departure timestamps) were missing (*n* = 1240), ED LOS was negative or otherwise implausible due to time-value or formatting errors (*n* = 8765), ED disposition (e.g., discharge, admission, transfer, or death) was missing (*n* = 1104), or (4) diagnostic code information was insufficient to derive the primary exposure, the ICD-based Injury Severity Score (ICISS), resulting in inability to calculate ICISS (*n* = 1982). After applying these criteria, the final analytic sample included 1,035,484 patients. Special situations in which interpretation of ED LOS may differ from typical patient flow (e.g., dead on arrival) were handled separately in sensitivity analyses or excluded in accordance with the NEDIS codebook. A flow diagram of record selection and exclusions is presented in Figure 1.

### 2.3. Data Collection

This study did not collect primary data through surveys or clinical trials. Instead, it constructed an analytic dataset using de-identified NEDIS raw data, constituting a secondary data analysis. The primary outcome, ED LOS, was calculated by combining emergency department arrival and departure date-time variables and was converted into minutes or hours for analysis. The primary exposure was defined as ICISS, a diagnosis-code–based injury severity measure; the primary analysis used the ICISS version based on 15 diagnoses, and a sensitivity analysis additionally applied the ICISS version based on 20 diagnoses.

The mediator was emergency department disposition, which was recategorized into three categories—discharge, admission, and transfer—based on the original disposition codes; binary indicators for admission and transfer were also created as needed for specific analyses. Covariates included demographic characteristics (age and sex), injury characteristics (mechanism of injury and intentionality), visit characteristics (route of presentation and mode of arrival), and initial clinical status (level of consciousness and vital signs). During data cleaning, values indicating “not applicable” or “not recorded” were treated as missing. Given the potential for missingness in variables such as vital signs, missing-data handling strategies (e.g., complete-case analysis, missing-indicator methods, and multiple imputation) were specified a priori, and sensitivity analyses were performed to assess the robustness of findings.

### 2.4. Statistical Analysis

All analyses were conducted using validated statistical software such as IBM SPSS Statistics (version 27; IBM Corp., Armonk, NY, USA), and statistical significance was defined as a two-sided *p* value < 0.05. Descriptive statistics were first used to summarize the distribution of ED LOS, reporting the median and interquartile range given its likely right-skewed distribution. Distributions of ICISS, disposition (discharge, admission, transfer), and other covariates were also described.

To identify determinants of ED LOS, a gamma regression model with a log link was used as the primary analytical approach, reflecting the positive and right-skewed nature of ED LOS. A log-transformed linear regression model was also fitted as an alternative model to assess consistency of results. Covariate adjustment was performed using sequential models to compare changes in the estimated ICISS effect across steps, including adjustment for demographic variables, initial clinical status, injury and visit characteristics, and inclusion of disposition. In particular, changes in the ICISS coefficient before and after inclusion of disposition were examined to explore the extent to which the association between injury severity and ED LOS may be explained through admission or transfer pathways.

The primary inferential analysis applied causal mediation analysis to quantify the indirect effect of ICISS (exposure) on ED LOS (outcome) mediated through disposition (admission and transfer). Specifically, a mediator model (disposition ~ ICISS + covariates) and an outcome model (ED LOS ~ ICISS + disposition + covariates) were fitted, and the average causal mediation effect, average direct effect, and total effect were estimated with 95% confidence intervals derived using bootstrap resampling. For greater interpretability, separate mediation analyses were conducted treating admission and transfer as distinct mediators, and the results were presented in parallel.

Additional analyses compared the consistency of findings between ICISS based on 15 diagnoses and ICISS based on 20 diagnoses and conducted sensitivity analyses for handling extreme ED LOS values (e.g., exclusion based on predefined criteria and winsorization) and missing data (complete-case analysis versus missing-indicator methods or multiple imputation). Where appropriate, interaction analyses (e.g., ICISS by ambulance arrival) and age-stratified analyses were also performed to assess whether the relationships among injury severity, disposition, and ED LOS differed across subgroups.

## 3. Results

### 3.1. General Characteristics

The participants’ general characteristics are summarized in Table 1. The analytic sample included 1,035,484 injured patients: 807,678 (78.0%)were discharged, 186,387 (18.0%) were admitted, and 41,419 (4.0%)were transferred. Patients in the admission and transfer groups were older than those discharged (mean age 55.6 and 49.3 vs. 42.1 years), and males accounted for approximately 60%overall. Falls and traffic accidents were the most common injury mechanisms, with falls more prevalent among admissions and transfers, whereas traffic accidents were relatively more common among discharges. Most injuries were unintentional (85.2%) across all groups.

Marked differences were observed in care pathways: transferred-in visits were uncommon among discharges (8.0%) but substantially higher among admissions (25.0%) and especially transfers (65.0%). Similarly, ambulance/EMS arrival was more frequent in admissions (55.0%) and transfers (65.0%) than in discharges (30.0%). Initial condition also differed: non-alert mental status and less favorable vital signs were more common in the admission and transfer groups. Injury severity showed the clearest gradient—mean ICISSwas highest in discharges (0.991) and lower in admissions (0.972) and transfers (0.965); the proportion with ICISS <0.95 increased from 3.0% (discharge) to 12.0% (admission) and 18.0% (transfer) (Table 1).

### 3.2. Distribution of ED Length of Stay

Among 1,035,484 injured patients, the overall median ED length of stay (ED LOS) was 150 min (IQR 90–260; 2.5 h [IQR 1.5–4.3]). ED LOS differed markedly by disposition, with substantially longer stays in the admission and transfer groups than in the discharge group (median 420 min [7.0 h] and 360 min [6.0 h] vs. 120 min [2.0 h], respectively). The upper tail of ED LOS was pronounced, reaching 10 h (90th), 18 h (95th), and 48 h (99th) overall, and was most extreme among admitted patients (99th percentile 72 h).

Prolonged ED LOS was observed in 17.9% of all visits for ≥6 h, 6.9% for ≥12 h, 2.5% for ≥24 h, and 0.7% for ≥48 h. Prolonged stays were concentrated in the admission and transfer pathways: 55.0% and 45.0% of these patients remained in the ED for ≥6 h (vs. 8.0% in the discharge group), and 25.0% and 20.0% remained for ≥12 h (vs. 2.0%), respectively (Table 2) (Appendix A.1).

### 3.3. Factors Associated with ED LOS with Gamma Regression with Log Link

Table 3 summarizes the factors associated with emergency department (ED) length of stay (LOS) using gamma regression with a log link. In the unadjusted model (Model 1), greater injury severity (per 0.05 decrease in ICISS) was strongly associated with longer ED LOS (Ratio 1.34, 95% CI 1.33–1.35; *p* < 0.001). This association remained statistically significant after adjustment for demographics (Model 2; Ratio 1.31, 95% CI 1.30–1.32; *p* < 0.001) and further adjustment for visit and injury characteristics and initial clinical status (Model 3; Ratio 1.26, 95% CI 1.25–1.27; *p* < 0.001). After additional adjustment for disposition (Model 4), the magnitude of the association was attenuated but remained significant (Ratio 1.12, 95% CI 1.11–1.13; *p* < 0.001).

In Models 2–4, older age was associated with longer ED LOS (Model 4: per 10-year increase, Ratio 1.02, 95% CI 1.02–1.03; *p* < 0.001). Female sex showed a small association in partially adjusted models (Model 2–3), but was not significant in the fully adjusted model (Model 4: Ratio 1.00, 95% CI 0.99–1.01; *p* = 0.64). In Model 3 and Model 4, ambulance arrival (Model 4: Ratio 1.07, 95% CI 1.06–1.08; *p* < 0.001) and transferred-in status (Model 4: Ratio 1.23, 95% CI 1.21–1.25; *p* < 0.001) were associated with longer ED LOS. Traffic-related mechanism (Model 4: Ratio 1.03, 95% CI 1.02–1.04; *p* < 0.001) and self-harm intent (Model 4: Ratio 1.05, 95% CI 1.02–1.08; *p* = 0.002) were also associated with increased LOS. Indicators of worse initial physiologic/neurologic status were consistently associated with prolonged LOS, including non-alert mental status (Model 4: Ratio 1.11, 95% CI 1.09–1.13; *p* < 0.001), lower systolic blood pressure (per 10 mmHg decrease; Ratio 1.01, 95% CI 1.01–1.02; *p* < 0.001), and higher heart rate (per 10 bpm increase; Ratio 1.01, 95% CI 1.01–1.02; *p* < 0.001).

Disposition variables were the strongest correlates of ED LOS in Model 4: admission was associated with more than a two-fold longer LOS (Ratio 2.35, 95% CI 2.32–2.38; *p* < 0.001), and inter-hospital transfer was similarly associated with longer LOS (Ratio 2.05, 95% CI 2.00–2.10; *p* < 0.001), suggesting that output-stage processes substantially contribute to prolonged ED stays among injured patients (Table 3).

### 3.4. Factors Associated with Admission and Transfer in Mediation Models

Table 4 presents the logistic regression results for factors associated with admission and inter-hospital transfer among injured patients. Greater injury severity was strongly associated with both outcomes: for each 0.05 decrease in ICISS, the odds of admission increased approximately two-fold (OR 2.10, 95% CI 2.07–2.13; *p* < 0.001) and the odds of transfer increased even more (OR 2.45, 95% CI 2.38–2.52; *p* < 0.001). Older age was also associated with higher odds of admission (per 10-year increase, OR 1.28, 95% CI 1.27–1.29; *p* < 0.001) and transfer (OR 1.15, 95% CI 1.14–1.16; *p* < 0.001). Female sex was associated with slightly lower odds of admission (OR 0.92, 95% CI 0.91–0.94; *p* < 0.001) and transfer (OR 0.95, 95% CI 0.93–0.98; *p* < 0.001).

Mode of arrival and referral status were significant predictors. Ambulance arrival was associated with higher odds of admission (OR 2.05, 95% CI 2.02–2.09; *p* < 0.001) and transfer (OR 1.78, 95% CI 1.72–1.84; *p* < 0.001). Patients transferred in from another hospital had increased odds of admission (OR 1.60, 95% CI 1.58–1.63; *p* < 0.001) and markedly increased odds of subsequent transfer (OR 6.20, 95% CI 6.02–6.39; *p* < 0.001).

Injury intent and mechanism showed differential associations. Traffic-related injuries were associated with lower odds of admission (OR 0.84, 95% CI 0.83–0.86; *p* = 0.006) but higher odds of transfer (OR 1.12, 95% CI 1.09–1.15; *p* < 0.001). Self-harm was associated with higher odds of admission (OR 1.35, 95% CI 1.29–1.41; *p* = 0.021), whereas its association with transfer was not statistically significant (OR 0.95, 95% CI 0.88–1.03; *p* = 0.22).

Clinical status at presentation was consistently associated with disposition. Non-alert mental status was strongly associated with admission (OR 1.75, 95% CI 1.71–1.80; *p* < 0.001) and transfer (OR 2.30, 95% CI 2.20–2.41; *p* < 0.001). Lower systolic blood pressure (per 10 mmHg decrease) increased the odds of admission (OR 1.12, 95% CI 1.11–1.13; *p* < 0.001) and transfer increase (OR 1.15, 95% CI 1.13–1.17; *p* < 0.001), and higher heart rate (per 10 bpm increase) was also associated with both admission (OR 1.07, 95% CI 1.06–1.08; *p* < 0.001) and transfer (OR 1.09, 95% CI 1.08–1.11; *p* < 0.001) (Table 4).

### 3.5. Causal Mediation Analysis Results: Disposition Mediating the ICISS–ED LOS Association

In the mediation analysis, a 0.05 decrease in ICISS is associated with a total effect on ED LOS of 0.231 (95% CI 0.228–0.234, *p* < 0.001). When admission is specified as the mediator, the average causal mediation effect (ACME; indirect effect) is 0.085 (95% CI 0.083–0.087, *p* < 0.001) and the average direct effect (ADE) is 0.146 (95% CI 0.143–0.149, *p* < 0.001), with admission mediating 36.8% of the total effect. When transfer is specified as the mediator, the ACME is 0.033 (95% CI 0.031–0.035, *p* < 0.001) and the ADE is 0.198 (95% CI 0.195–0.201, *p* < 0.001), with transfer mediating 14.3% of the total effect (Table 5).

### 3.6. Sensitivity and Robustness Analyses

Across sensitivity analyses, the association between injury severity and ED LOS remains consistent. In the primary analysis using ICISS based on 15 diagnoses, a 0.05 decrease in ICISS is associated with a longer ED LOS (Ratio 1.12, 95% CI 1.11–1.13), with admission mediating 36.8% of the effect (ACME 0.085) and transfer mediating 14.3% (ACME 0.033). Using an alternative severity metric (ICISS based on 20 diagnoses) yields similar estimates (Ratio 1.11, 95% CI 1.10–1.12), with 35.9% of the effect mediated by admission and 13.9% mediated by transfer.

When outliers are excluded according to predefined rules, the ICISS effect remains unchanged (Ratio 1.11, 95% CI 1.10–1.12), and the mediated proportions are 37.5% for admission and 14.6% for transfer. Winsorizing ED LOS at the top 1% slightly attenuates the ICISS effect (Ratio 1.10, 95% CI 1.09–1.11) while maintaining comparable mediation (38.2% via admission; 15.1% via transfer). Complete-case analysis produces similar results (Ratio 1.13, 95% CI 1.12–1.14), with 36.1% mediated by admission and 14.0% by transfer. Approaches to missing data yield consistent findings, including the missing-indicator method (Ratio 1.12, 95% CI 1.11–1.13; 36.7% and 14.2% mediated by admission and transfer, respectively) and multiple imputation (Ratio 1.12, 95% CI 1.11–1.13; 36.5% and 14.1% mediated).

**Figure 2:**
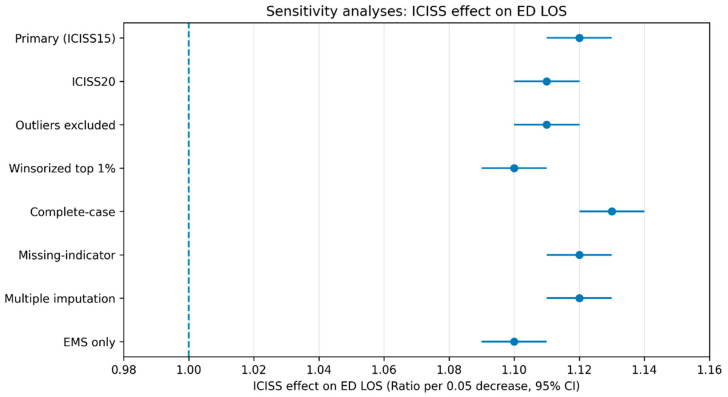
Sensitivity Analyses of the Association Between Injury Severity (ICISS) and ED Length of Stay. Blue dots indicate point estimates and blue horizontal lines indicate 95% confidence intervals. The dashed vertical line at 1.00 represents the null effect (no association).

Results are also robust to model specification. In a log-linear regression framework, a 0.05 decrease in ICISS corresponds to β = 0.113 (95% CI 0.104–0.122), with comparable indirect effects (Admission ACME 0.084; Transfer ACME 0.032). In the subgroup restricted to patients arriving by ambulance/EMS, the association persists (Ratio 1.10, 95% CI 1.09–1.11), with admission mediating 34.8% and transfer mediating 15.7% of the effect (Table 6) (Figure 2) (Appendix A.2 and Appendix A.3).

## 4. Discussion

This study, using nationwide NEDIS data collected from the date of Institutional Review Board (IRB) approval through 12 January 2026, examined 1,035,484 injured patients to describe the distribution and determinants of emergency department length of stay (ED LOS) and to evaluate the extent to which the effect of injury severity (ICISS) on ED LOS is mediated through clinical disposition (admission and inter-hospital transfer). ED LOS is regarded as a representative throughput metric within the input–throughput–output model of patient flow; however, it is well recognized that ED LOS is strongly influenced by output-stage constraints such as inpatient bed availability, boarding, and transfer coordination [16]. Prior studies have comprehensively summarized the causes, consequences, and solutions to ED crowding and have repeatedly highlighted admission delays and hospital system factors as key drivers of prolonged ED LOS [17,18]. Nevertheless, relatively few studies have explicitly decomposed the severity–ED LOS relationship into unavoidable prolongation attributable to severity-related clinical processes and output-stage delays arising during admission and transfer, while simultaneously quantifying the contribution of each pathway to the overall effect. In this context, our findings provide clinically, operationally, and policy-relevant implications.

First, the distribution of ED LOS was characteristically right-skewed with a long tail, with an overall median of 150 min (2.5 h). Marked differences were observed by disposition: the median ED LOS was 120 min (2.0 h) among discharged patients, compared with 420 min (7.0 h) among admitted patients and 360 min (6.0 h) among transferred patients. Moreover, prolonged ED LOS accounted for a substantial proportion of visits, with 17.9% staying ≥ 6 h, 6.9% staying ≥ 12 h, and 2.5% staying ≥ 24 h, indicating that structurally prolonged stays occur in a meaningful subset of patients. Notably, prolonged ED LOS was concentrated among patients who were admitted or transferred: the proportion staying ≥ 6 h was 55.0% among admitted patients and 45.0% among transferred patients, far exceeding that among discharged patients (8.0%). These findings are consistent with prior observations that ED crowding cannot be explained solely by the efficiency of internal ED processing (throughput), and that hospital-wide “access block” driven by inpatient bed shortages and boarding is a primary mechanism underlying prolonged ED LOS [19]. Evidence has long accumulated demonstrating that higher hospital occupancy is associated with significantly longer ED LOS among patients awaiting admission [19], which directly aligns with our finding that admitted patients had the longest ED LOS.

In addition, hospital size and system capacity likely shape ED crowding and prolonged LOS through access block and queueing dynamics. Larger hospitals frequently operate under higher demand intensity and greater variability in arrivals, which can elevate average occupancy and amplify congestion during peak periods. From a queueing perspective, as utilization approaches capacity, waiting times may rise nonlinearly, and operational responses such as diversion or “turn-away” can emerge—phenomena commonly conceptualized using Erlang-type models. Although the present NEDIS raw dataset is de-identified and does not include hospital identifiers or hospital-level capacity/occupancy metrics to directly test these mechanisms, the strong associations of admission and inter-hospital transfer with prolonged ED LOS observed in this study are consistent with an output-stage bottleneck framework. Future linkage of visit-level ED data to hospital-level operational indicators (e.g., annual ED volume, bed capacity, occupancy, diversion/ambulance offload delays) would allow quantification of between-hospital heterogeneity and improve the generalizability of these findings beyond Korea.

Second, the association between injury severity (ICISS) and ED LOS was consistently observed across all models. A 0.05 decrease in ICISS (i.e., increased severity) was associated with a 1.34-fold longer ED LOS in the unadjusted model, and the association remained statistically significant after sequential adjustment for demographic characteristics, visit-related factors, initial physiological status, injury-related variables, and disposition, with a 1.12-fold longer ED LOS in the fully adjusted model. This pattern reflects a “severity-specific effect,” whereby more severe injuries require additional time for initial stabilization, imaging and laboratory testing, procedures and interventions, consultations, and decisions regarding surgery or intensive care admission. At the same time, attenuation of the effect size after adjustment suggests that factors that co-occur with higher severity (e.g., ambulance transport, transfer-in presentation, altered mental status, and abnormal vital signs) also contribute to prolonged ED LOS, indicating that the severity–LOS relationship cannot be reduced to a single mechanism. This is consistent with national evidence from Korea showing that prolonged ED LOS results from the combined influence of patient characteristics and operational factors [20].

Importantly, a key finding of this study is that admission and transfer not only substantially prolong ED LOS but also function as major pathways through which severity exerts its effect. In the fully adjusted model, admission and transfer were associated with 2.35-fold and 2.05-fold increases in ED LOS, respectively, confirming that core drivers of prolonged ED stays are closely linked to disposition. This is concordant with prior studies suggesting that boarding after the admission decision may be associated not only with lower patient satisfaction but also with longer inpatient length of stay and increased mortality risk [20]. Although the strength of the association between boarding and mortality varies across study designs and populations, systematic reviews have summarized that a clear safety threshold may not exist and that an overall unfavorable trend is often observed [21]. Nevertheless, given domestic evidence indicating that prolonged ED LOS is associated with adverse events among critically ill patients (e.g., in-hospital cardiac arrest) [22], delays in admission and transfer remain important targets for improving patient safety among severely injured patients.

The primary contribution of this study is the quantification of the mediating role of disposition in the relationship between injury severity (ICISS) and ED LOS. Our mediation analysis showed that admission mediated 36.8% and transfer mediated 14.3% of the severity–ED LOS association. This suggests that while a substantial portion of prolonged ED LOS among severely injured patients reflects the direct effect of clinically necessary processes, a comparable share is explained by output-stage bottlenecks—namely, inpatient bed assignment/boarding and the search, coordination, and arrangement of receiving hospitals for transfer—which indirectly extend ED LOS [23]. The larger mediating proportion for admission compared with transfer further implies that, at the national level, reducing prolonged ED LOS may require not only strengthening transfer systems but also prioritizing inpatient bed operations and boarding management as high-leverage interventions. This aligns with conclusions from prior systematic reviews that ED crowding cannot be adequately addressed through ED process improvements alone and requires hospital-wide system-level approaches [17,18].

The robustness of our findings across sensitivity analyses further strengthens the credibility of the conclusions. The direction and magnitude of the associations between severity and ED LOS, as well as the mediation effects, remained broadly similar when using alternative ICISS definitions (e.g., based on 20 diagnoses), excluding extreme values or applying winsorization, and employing different strategies for handling missing data or alternative model specifications. This indicates that the results are unlikely to be driven by a small number of extreme ED LOS observations or by a particular modeling choice, and instead likely reflect structural features of injury care flow in the ED.

These findings yield several practical and policy implications. First, the most critical targets for reducing prolonged ED LOS among injured patients are those requiring admission or transfer, with particular emphasis on alleviating admission-stage bottlenecks through hospital-level strategies such as bed management, early bed assignment, and boarding reduction initiatives [19]. Second, because transfer-related delays account for a nontrivial portion of prolonged ED LOS, improving regional resource visibility (e.g., ICU capacity and surgical availability), standardizing transfer protocols, and strengthening transfer coordination functions may contribute to ED LOS improvements [24]. In Korea, reports indicating that not only single transfers but also double inter-hospital transfers affect utilization patterns and burdens in higher-acuity EDs support the need for more refined regionalization and transfer systems [25]. Third, because ED LOS is associated not only with operational efficiency but also with patient safety [26], balanced strategies are needed that preserve clinically necessary time for care while minimizing avoidable output-stage waiting.

Several limitations should be noted. First, this was an observational study, so residual unmeasured confounding may remain in the relationships among injury severity, disposition, and ED length of stay, and the mediation estimates should be interpreted as quantitative evidence consistent with partial transmission through admission and inter-hospital transfer rather than definitive causal effects. Second, ED length of stay was calculated from recorded arrival and departure timestamps, and documentation errors or variability in data quality may persist. Third, the injury severity measure based on diagnosis codes may not fully capture physiologic severity or detailed anatomic injury information and may be influenced by coding practices. Fourth, the de-identified dataset did not include hospital identifiers or hospital-level operational measures such as ED volume, bed capacity or occupancy, staffing, imaging capacity, or trauma system designation, which precluded assessment of between-hospital heterogeneity and specific sources of output-stage bottlenecks. Fifth, limited date information in the analytic dataset prevented evaluation of seasonal variation in ED length of stay. Finally, disposition was categorized as discharge, admission, and transfer, and more granular pathways within admission could not be examined.

Future work should link visit-level NEDIS records to hospital-level datasets describing annual ED volume and capacity, and to operational measures such as bed occupancy and diversion/ambulance offload delays.

In conclusion, this nationwide study of injured patients demonstrated that ED LOS has a right-skewed distribution with a long tail and that prolonged stays are concentrated among patients who are admitted or transferred. Injury severity (ICISS) was independently associated with longer ED LOS, and a substantial portion of this association was mediated through admission (approximately 36.8%) and transfer (approximately 14.3%) pathways. These findings suggest that reducing ED LOS among severely injured patients requires not only efforts to streamline clinical processes but also system-level interventions aimed at mitigating output-stage bottlenecks, including inpatient bed operations, boarding management, and transfer coordination.

## 5. Conclusions

Our findings showed that ED length of stay (ED LOS) among injured patients had a right-skewed, asymmetric distribution with a median of 150 min. By disposition, ED LOS increased markedly from 120 min among discharged patients to 420 min among admitted patients and 360 min among transferred patients, and prolonged stays were concentrated in the admission and transfer pathways. In addition, a 0.05 decrease in ICISS (i.e., greater injury severity) was significantly associated with longer ED LOS even after covariate adjustment (adjusted ratio, 1.12), confirming that injury severity is an independent determinant of prolonged ED LOS. Admission and transfer were key factors substantially increasing ED LOS (2.35-fold for admission and 2.05-fold for transfer), and they functioned as major pathways through which the severity effect was transmitted. Mediation analysis indicated that admission mediated 36.8% and transfer mediated 14.3% of the association between injury severity and ED LOS, suggesting that prolonged ED LOS in severely injured patients is explained not only by clinically necessary diagnostic and treatment processes but also to a considerable extent by output-stage bottlenecks such as bed assignment/boarding and transfer coordination. Therefore, reducing ED LOS among severely injured patients requires a combined strategy that streamlines diagnostic and treatment processes while implementing system-level interventions to improve inpatient bed operations and boarding management and to strengthen transfer coordination.

## Figures and Tables

**Table 1:** General characteristics of survey participants (*n* = 1,035,484).

Variables	Overall (*n* = 1,035,484)	Discharge (*n* = 807,678)	Admission (*n* = 186,387)	Transfer (*n* = 41,419)
Age, years	44.8 (19.7)	42.1 (18.6)	55.6 (20.4)	49.3 (21.1)
Sex				
Male, n (%)	619,220 (59.8)	476,530 (59.0)	117,424 (63.0)	25,266 (61.0)
Female, n (%)	416,264 (40.2)	331,148 (41.0)	68,963 (37.0)	16,153 (39.0)
Mechanism of injury				
Traffic accident, n (%)	333,634 (32.2)	274,611 (34.0)	46,597 (25.0)	12,426 (30.0)
Fall, n (%)	294,698 (28.5)	201,919 (25.0)	78,282 (42.0)	14,497 (35.0)
Penetrating injury, n (%)	39,556 (3.8)	32,307 (4.0)	5592 (3.0)	1657 (4.0)
Assault/violence, n (%)	78,696 (7.6)	64,614 (8.0)	11,183 (6.0)	2899 (7.0)
Other, n (%)	288,900 (27.9)	234,227 (29.0)	44,733 (24.0)	9940 (24.0)
Intentionality				
Unintentional, n (%)	882,439 (85.2)	694,603 (86.0)	154,701 (83.0)	33,135 (80.0)
Self-harm/suicide attempt, n (%)	22,574 (2.2)	16,154 (2.0)	5592 (3.0)	828 (2.0)
Violence/assault, n (%)	75,590 (7.3)	56,537 (7.0)	14,911 (8.0)	4142 (10.0)
Unknown, n (%)	54,881 (5.3)	40,384 (5.0)	11,183 (6.0)	3314 (8.0)
Route of presentation				
Direct visit, n (%)	897,351 (86.6)	743,064 (92.0)	139,790 (75.0)	14,497 (35.0)
Transferred in, n (%)	138,133 (13.4)	64,614 (8.0)	46,597 (25.0)	26,922 (65.0)
Mode of arrival				
Ambulance (119/EMS), n (%)	371,738 (35.9)	242,303 (30.0)	102,513 (55.0)	26,922 (65.0)
Walk-in/other, n (%)	663,746 (64.1)	565,375 (70.0)	83,874 (45.0)	14,497 (35.0)
Initial mental status				
Alert, n (%)	942,705 (91.0)	751,141 (93.0)	158,429 (85.0)	33,135 (80.0)
Verbal, n (%)	53,224 (5.1)	32,307 (4.0)	16,775 (9.0)	4142 (10.0)
Pain, n (%)	31,064 (3.0)	20,192 (2.5)	8387 (4.5)	2485 (6.0)
Unresponsive, n (%)	8491 (0.8)	4038 (0.5)	2796 (1.5)	1657 (4.0)
Initial vital signs				
SBP, mmHg, Mean (SD)	129.4 (22.8)	131.2 (21.5)	121.8 (26.9)	118.6 (28.4)
HR, bpm, Mean (SD)	86.7 (18.9)	84.9 (18.1)	92.8 (20.7)	96.4 (22.3)
RR,/min, Mean (SD)	18.7 (4.6)	18.3 (4.3)	20.1 (5.2)	20.8 (5.6)
SpO_2_, %, Mean (SD)	97.1 (2.9)	97.5 (2.5)	96.0 (3.6)	95.5 (4.1)
Temperature, °C, Mean (SD)	36.6 (0.6)	36.6 (0.5)	36.7 (0.6)	36.7 (0.7)
Injury severity (ICISS)				
ICISS (15 diagnoses), Mean (SD)	0.987 (0.018)	0.991 (0.012)	0.972 (0.028)	0.965 (0.035)
ICISS category n, (%)				
<0.95	54,051 (5.2)	24,230 (3.0)	22,366 (12.0)	7455 (18.0)
0.95–0.99	470,111 (45.4)	339,225 (42.0)	108,105 (58.0)	22,781 (55.0)
≥0.99	511,322 (49.4)	444,223 (55.0)	55,916 (30.0)	11,183 (27.0)

Data are presented as *n* (%) or mean (standard deviation). ICISS indicates the International Classification of Diseases-based Injury Severity Score (calculated using up to 15 diagnosis codes). SBP indicates systolic blood pressure; HR, heart rate; RR, respiratory rate; SpO_2_, peripheral oxygen saturation; EMS, emergency medical services.

**Table 2:** Distribution of ED length of stay (ED LOS).

ED LOS Metrics	Overall (*n* = 1,035,484)	Discharge (*n* = 807,678)	Admission (*n* = 186,387)	Transfer (*n* = 41,419)	*p* Value
ED LOS, minutes, Median (IQR)	150 (90–260)	120 (75–190)	420 (240–720)	360 (210–600)	<0.001
ED LOS, hours, Median (IQR)	2.5 (1.5–4.3)	2.0 (1.3–3.2)	7.0 (4.0–12.0)	6.0 (3.5–10.0)	<0.001
90th percentile, hours	10	5	24	18	—
95th percentile, hours	18	8	36	30	—
99th percentile, hours	48	18	72	60	—
Prolonged ED LOS					
≥6 h, n (%)	185,765 (17.9)	64,614 (8.0)	102,513 (55.0)	18,638 (45.0)	<0.001
≥12 h, n (%)	71,035 (6.9)	16,154 (2.0)	46,597 (25.0)	8284 (20.0)	<0.001
≥24 h, n (%)	25,991 (2.5)	4038 (0.5)	18,639 (10.0)	3314 (8.0)	<0.001
≥48 h (optional), n (%)	7228 (0.7)	808 (0.1)	5592 (3.0)	828 (2.0)	<0.001

ED LOS is presented as median (interquartile range, IQR) unless otherwise specified. Prolonged ED LOS is presented as *n* (%). *p* values for ED LOS medians and prolonged ED LOS categories are based on the Kruskal–Wallis test and χ^2^ test, respectively. Percentile values (90th/95th/99th) are descriptive summaries and were not formally tested. ED LOS indicates emergency department length of stay; ED, emergency department; IQR, interquartile range.

**Table 3:** Factors associated with ED LOS (Gamma regression with log link).

Variable	Model 1 Ratio (95% CI)	*p* Value	Model 2 Ratio (95% CI)	*p* Value	Model 3 Ratio (95% CI)	*p* Value	Model 4 Ratio (95% CI)	*p* Value
ICISS (per 0.05 decrease)	1.34 (1.33–1.35)	<0.001	1.31 (1.30–1.32)	<0.001	1.26 (1.25–1.27)	<0.001	1.12 (1.11–1.13)	<0.001
Age (per 10-year increase)	—	—	1.05 (1.05–1.06)	<0.001	1.04 (1.04–1.05)	<0.001	1.02 (1.02–1.03)	<0.001
Female (vs. Male)	—	—	0.98 (0.97–0.99)	<0.001	0.99 (0.98–1.00)	0.032	1.00 (0.99–1.01)	0.64
Ambulance arrival (vs. other)	—	—	—	—	1.18 (1.17–1.19)	<0.001	1.07 (1.06–1.08)	<0.001
Transferred in (vs. direct)	—	—	—	—	1.42 (1.40–1.44)	<0.001	1.23 (1.21–1.25)	<0.001
Mechanism: Traffic accident	—	—	—	—	1.06 (1.05–1.07)	<0.001	1.03 (1.02–1.04)	<0.001
Intentionality: Self-harm	—	—	—	—	1.10 (1.07–1.13)	<0.001	1.05 (1.02–1.08)	0.002
Initial mental status (non-alert vs. alert)	—	—	—	—	1.24 (1.22–1.26)	<0.001	1.11 (1.09–1.13)	<0.001
SBP (per 10 mmHg decrease)	—	—	—	—	1.03 (1.03–1.04)	<0.001	1.01 (1.01–1.02)	<0.001
HR (per 10 bpm increase)	—	—	—	—	1.02 (1.02–1.03)	<0.001	1.01 (1.01–1.02)	<0.001
Disposition included (Model 4 only)								
Admission (yes vs. no)	—	—	—	—	—	—	2.35 (2.32–2.38)	<0.001
Transfer (yes vs. no)	—	—	—	—	—	—	2.05 (2.00–2.10)	<0.001

Ratios are exponentiated coefficients from gamma regression models with a log link for ED LOS and are reported as Ratio with 95% confidence interval. ICISS is scaled per 0.05 decrease, indicating greater injury severity. Age is scaled per 10-year increase. SBP is scaled per 10 mmHg decrease and HR per 10 bpm increase. Non-alert indicates any mental status other than alert, including verbal, pain, or unresponsive. Model 1 includes ICISS only. Model 2 additionally adjusts for age and sex. Model 3 further adjusts for ambulance arrival, transferred-in status, mechanism defined as traffic accident, intentionality defined as self-harm, initial mental status, SBP, and HR. Model 4 additionally adjusts for disposition variables, including admission and inter-hospital transfer. ED LOS indicates emergency department length of stay. ICISS indicates International Classification of Disease-based Injury Severity Score. SBP indicates systolic blood pressure. HR indicates heart rate. CI indicates confidence interval.

**Table 4:** Mediator models: factors associated with admission and transfer.

Variable	Admission OR (95% CI)	*p* Value	Transfer OR (95% CI)	*p* Value
ICISS (per 0.05 decrease)	2.10 (2.07–2.13)	<0.001	2.45 (2.38–2.52)	<0.001
Age (per 10-year increase)	1.28 (1.27–1.29)	<0.001	1.15 (1.14–1.16)	<0.001
Female (vs. Male)	0.92 (0.91–0.94)	<0.001	0.95 (0.93–0.98)	<0.001
Ambulance arrival (vs. other)	2.05 (2.02–2.09)	<0.001	1.78 (1.72–1.84)	<0.001
Transferred in (vs. direct)	1.60 (1.58–1.63)	<0.001	6.20 (6.02–6.39)	<0.001
Mechanism: Traffic accident	0.84 (0.83–0.86)	0.006	1.12 (1.09–1.15)	<0.001
Intentionality: Self-harm	1.35 (1.29–1.41)	0.021	0.95 (0.88–1.03)	0.22
Initial mental status (non-alert vs. alert)	1.75 (1.71–1.80)	<0.001	2.30 (2.20–2.41)	<0.001
SBP (per 10 mmHg decrease)	1.12 (1.11–1.13)	<0.001	1.15 (1.13–1.17)	<0.001
HR (per 10 bpm increase)	1.07 (1.06–1.08)	<0.001	1.09 (1.08–1.11)	<0.001

Odds ratios (ORs) and 95% confidence intervals (CIs) are from multivariable logistic regression models for (1) admission (yes vs. no) and (2) transfer (yes vs. no). ICISS is scaled per 0.05 decrease (greater injury severity). Age is scaled per 10-year increase. SBP is scaled per 10 mmHg decrease and HR per 10 bpm increase. “Non-alert” indicates any mental status other than alert (e.g., verbal/pain/unresponsive). Reference categories are male (for sex), non-ambulance arrival (for mode of arrival), direct visit (for route of presentation), and non–traffic accident/non–self-harm as applicable. ICISS indicates the International Classification of Diseases-based Injury Severity Score; SBP, systolic blood pressure; HR, heart rate; CI, confidence interval.

**Table 5:** Causal mediation analysis: disposition as mediator between ICISS and ED LOS.

Mediator	ACME (Indirect Effect) Estimate (95% CI)	*p* Value	ADE (Direct Effect) Estimate (95% CI)	*p* Value	Total Effect Estimate (95% CI)	*p* Value	Proportion Mediated (%)
Admission	0.085 (0.083–0.087)	<0.001	0.146 (0.143–0.149)	<0.001	0.231 (0.228–0.234)	<0.001	36.8
Transfer	0.033 (0.031–0.035)	<0.001	0.198 (0.195–0.201)	<0.001	0.231 (0.228–0.234)	<0.001	14.3

Mediation effects are reported as estimates (95% confidence intervals) from causal mediation analysis evaluating the effect of a 0.05 decrease in ICISS (greater injury severity) on ED LOS. ACME represents the average causal mediation effect (indirect effect through the mediator), ADE represents the average direct effect (effect not operating through the mediator), and the total effect equals ACME + ADE. The proportion mediated is calculated as ACME divided by the total effect and expressed as a percentage. Effect estimates are presented on the model link scale used for ED LOS (e.g., log scale for log-linked models). ICISS indicates the International Classification of Diseases-based Injury Severity Score; ED LOS, emergency department length of stay; CI, confidence interval.

**Table 6:** Sensitivity and robustness analyses.

Sensitivity Analysis	Main ICISS Effect on ED LOS (Ratio/β, 95% CI)	Key Mediation Result (ACME or % Mediated)	Summary Interpretation
Primary: ICISS (15 diagnoses)	Ratio 1.12 (1.11–1.13) per 0.05 decrease	Admission 36.8% mediated (ACME 0.085); Transfer 14.3% mediated (ACME 0.033)	Primary model shows a positive association; effects partly mediated by disposition.
Alternative severity: ICISS (20 diagnoses)	Ratio 1.11 (1.10–1.12)	Admission 35.9% mediated; Transfer 13.9% mediated	Results consistent when using ICISS20.
Outliers excluded (predefined rule)	Ratio 1.11 (1.10–1.12)	Admission 37.5% mediated; Transfer 14.6% mediated	Excluding implausible/outlier ED LOS does not materially change conclusions.
Winsorization of ED LOS (e.g., top 1%)	Ratio 1.10 (1.09–1.11)	Admission 38.2% mediated; Transfer 15.1% mediated	Trimming upper-tail ED LOS slightly attenuates the ICISS effect; mediation remains.
Complete-case analysis	Ratio 1.13 (1.12–1.14)	Admission 36.1% mediated; Transfer 14.0% mediated	Findings robust in complete cases.
Missing-indicator method	Ratio 1.12 (1.11–1.13)	Admission 36.7% mediated; Transfer 14.2% mediated	Handling missingness with indicators yields similar estimates.
Multiple imputation (if used)	Ratio 1.12 (1.11–1.13)	Admission 36.5% mediated; Transfer 14.1% mediated	Imputed analyses support the primary results.
Alternative model: log-linear regression	β = 0.113 (0.104–0.122) per 0.05 decrease	Admission ACME 0.084; Transfer ACME 0.032	Alternative functional form produces consistent direction and magnitude.
Subgroup: ambulance arrival only (optional)	Ratio 1.10 (1.09–1.11)	Admission 34.8% mediated; Transfer 15.7% mediated	Association persists among EMS-transported patients.

Sensitivity analyses evaluate the robustness of the association between injury severity (ICISS) and ED LOS and the corresponding mediation findings. The main ICISS effect is reported as a ratio (from gamma regression with a log link) per 0.05 decrease in ICISS, unless otherwise specified; for the log-linear regression, results are reported as β (95% CI) on the log scale. Key mediation results are summarized as the proportion mediated (%) and/or ACME (average causal mediation effect) for admission and transfer. “Outliers excluded” refers to removal of ED LOS values flagged by predefined data-cleaning rules; “Winsorization” truncates extreme ED LOS values at the upper tail (e.g., top 1%); “Complete-case” includes only records without missing covariates; the “Missing-indicator” method includes missingness indicators for covariates; and “Multiple imputation” uses imputed covariate values when applicable. The ambulance-arrival subgroup restricts analyses to patients transported by EMS. ICISS indicates the International Classification of Diseases-based Injury Severity Score; ED LOS, emergency department length of stay; ACME, average causal mediation effect; CI, confidence interval; EMS, emergency medical services.

**Figure A1:**
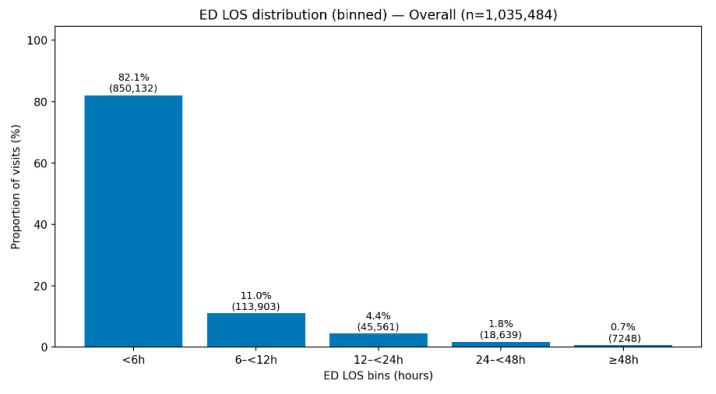
Distribution of emergency department length of stay (ED LOS) in the overall cohort (binned).

**Figure A2:**
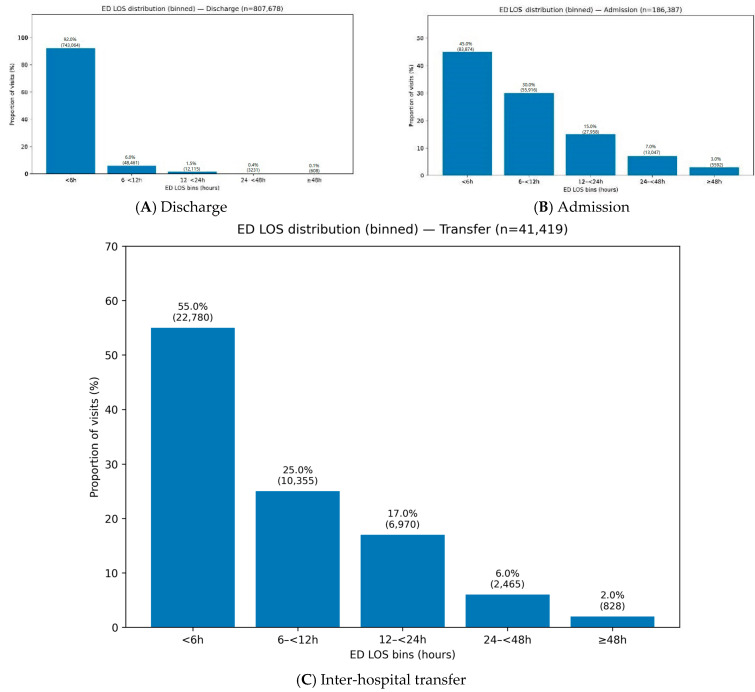
ED LOS distribution by disposition (binned).

**Figure A3:**
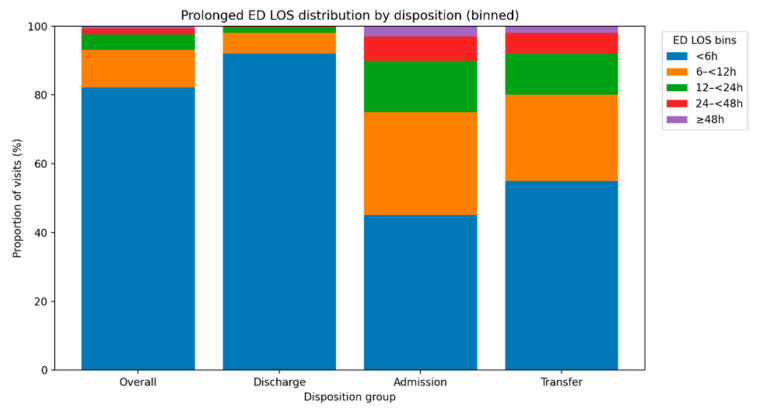
Prolonged ED LOS categories by disposition (stacked bar chart).

## Data Availability

The data used in this study are available from the National Emergency Department Information System (NEDIS); however, restrictions apply to the availability of these data, which were used under license/permission for the current study, and thus are not publicly available. Data may be available from the corresponding author upon reasonable request and with permission from the relevant data provider.

## Abbreviations

The following abbreviations are used in this manuscript:

ED	emergency department
ED LOS	emergency department length of stay
NEDIS	National Emergency Department Information System
ICISS	International Classification of Diseases-based Injury Severity Score
ICD	International Classification of Diseases
EMS	emergency medical services
OR	odds ratio
CI	confidence interval
ACME	average causal mediation effect

## Appendix A

### Appendix A.1

ED LOS is displayed as a binned distribution derived from the prolonged LOS thresholds reported in Table 2 (<6 h, 6– < 12 h, 12– < 24 h, 24– < 48 h, and ≥48 h). Bars represent the proportion of visits in each LOS bin; annotations indicate the corresponding percentages and counts. Total *n* = 1,035,484.

### Appendix A.2

ED LOS distributions are shown separately for (A) discharge (*n* = 807,678), (B) admission (*n* = 186,387), and (C) inter-hospital transfer (*n* = 41,419). LOS bins were derived from Table 2 prolonged LOS thresholds ( < 6 h, 6– < 12 h, 12– < 24 h, 24– < 48 h, and ≥48 h). Bars represent the proportion of visits in each LOS bin, with percentages and counts annotated.

### Appendix A.3

Stacked bars show the proportion of visits in each ED LOS bin (<6 h, 6– < 12 h, 12– < 24 h, 24– < 48 h, and ≥48 h) across the overall cohort and disposition subgroups (discharge, admission, and transfer), based on the prolonged LOS metrics reported in Table 2. This visualization highlights the greater concentration of prolonged LOS among admitted and transferred patients.
